# Data to model the prognosticators of luxury consumption: A partial least squares-structural equation modelling approach (PLS-SEM)

**DOI:** 10.1016/j.dib.2018.10.032

**Published:** 2018-10-15

**Authors:** Eugine Tafadzwa Maziriri, Nkosiville Welcome Madinga

**Affiliations:** a University of the Witwatersrand, South Africa; b University of Cape Town, South Africa

## Abstract

This article presents raw inferential statistical data that determined the impact of exclusivity, materialism, perceived quality and brand consciousness on luxury consumption. The data were collected from consumers within the Cape Town metropolitan area. A quantitative research method was used to analyse the data. Structured questionnaires were distributed to consumers within the Cape Town metropolitan area of South Africa. Reliability and validity were confirmed. Structural equation modelling (SEM) using the Smart PLS software, version 3, was used to present the data. The SEM path analysis shows the estimates of the interconnectedness of the major constructs in the data. The outcomes obtained from this dataset show the relationship between exclusivity, perceived quality and brand consciousness had a positive and a significant impact on luxury consumption. However, materialism proved to have a negative and insignificant influence on luxury consumption.

**Specifications table**

**Table:** 

Subject area	Marketing
Specific subject area	Consumer behaviour, apparel consumption
Type of data	Tables and figures
How data was acquired	Data was gathered significantly through the dissemination of questionnaires to clients inside the Cape Town Metropolitan region
Data format	Raw, analysed, descriptive and statistical data
Experimental factors	Sample consisted of consumers within the Cape Town metropolitan area.
	After inviting the consumers, the researcher-made questionnaires, which included demographic data and research questions pertaining to the variables under investigation, were completed.
	In this paper, the impact of exclusivity, materialism, perceived quality and brand consciousness on luxury consumption have been studied.
Experimental features	Factors such as exclusivity, materialism, perceived quality and brand consciousness are instrumental in stimulating luxury consumption
Data source location	Cape Town, South Africa
Data accessibility	Data is included in this article

**Value of the data**

• The data presents the impact of exclusivity, materialism, perceived quality, and brand consciousness on luxury consumption and shows the level of impact each of the variables have on each other.
• The data can be used as a springboard for further discourse on how marketing managers could enhance luxury consumption among consumers.
• The data can be modified for use in other contexts.
• The data can be used to enlighten marketing managers on the importance of exclusivity, materialism, perceived quality, and brand consciousness as well as how these four variables can be beneficial to increase luxury consumption among consumers.

## 1 Data

The data contained 157 usable copies of questionnaires retrieved from 170 copies administered to consumers in Cape Town, South Africa; hence, representing a response rate of 92%. First, the researchers engaged in the data preparation process. According to Aaker et al. [1], data preparation is regarded as a process of converting data from a questionnaire into a format that can be analysed. Furthermore, there are four phases of data preparation, namely data editing, coding, capturing and cleaning [1], [3]. These phases were employed to ensure that the data collected is complete and ready for analysing. After checking for missing values and outliers in the data, the researchers proceeded in assessing the reliability of test results. Three methods, namely Cronbach׳s alpha test (Cronbach α), composite reliability test (CR) and average value extracted (AVE) test were used in the study to check on the reliability of data. Table 1 below shows that all the reliability values were above the recommended value of 0.7 [4], suggesting excellent levels of internal consistency.

**Table 1:** Measurement accuracy assessment.

**Research constructs**	**PLS code item**	**Scale item**		**Cronbach׳s alpha value**	**Composite reliability**	**Average variance extracted (AVE)**	**Factor loadings**
		**Mean**	**SD**				
Materialism (M)	M2	3.178	1.181	0.789	0.845	0.481	0.663
	M3	3.930	0.932				0.690
	M4	3.439	0.986				0.764
	M5	3.611	1.081				0.666
	M6	3.522	1.026				0.518
	M7	3.159	1.154				0.823
Exclusivity (E)	E1	3.159	1.115	0.757	0.863	0.678	0.872
	E2	3.064	1.032				0.873
	E4	3.471	1.032				0.717
Perceived quality (PQ)	PQ1	3.274	1.104	0.776	0.849	0.586	0.858
	PQ2	3.140	1.202				0.760
	PQ3	3.873	0.949				0.674
	PQ4	3.535	1.080				0.761
Brand consciousness (BC)	BC1	3.395	1.081	0.798	0.850	0.489	0.598
	BC3	3.541	1.056				0.658
	BC5	3.414	0.978				0.831
	BC6	3.573	1.060				0.707
	BC7	3.497	0.988				0.618
	BC8	3.025	1.094				0.753
Luxury consumption (LC)	LC1	3.013	1.041	0.813	0.878	0.643	0.856
	LC2	3.025	1.022				0.802
	LC4	3.331	0.967				0.697
	LC5	3.089	1.055				0.844

## 2 Experimental design, materials, and methods

The data presented was based on a quantitative study. A descriptive research design was adopted in this study to obtain the opinions of customers concerning the prognosticators of luxury consumption. A survey method was considered an appropriate data collection method because it allows for the collection of standardised data that permits the researcher to produce information for answering the how, who, what, and when questions regarding the subject matter. Customers within the Cape Town metropolitan area were selected for the study. To test the data, the researchers proposed the model whereby exclusivity, materialism, perceived quality and brand consciousness were the predictor variables and luxury consumption was the outcome variable. The researchers had to propose a model to test the validity of the proposed model as well as to determine if the data, which has been collected in the field, fits well with the proposed conceptual model.

### 2.1 Assessment of the goodness of fit (GOF)

**Fig. 1:**
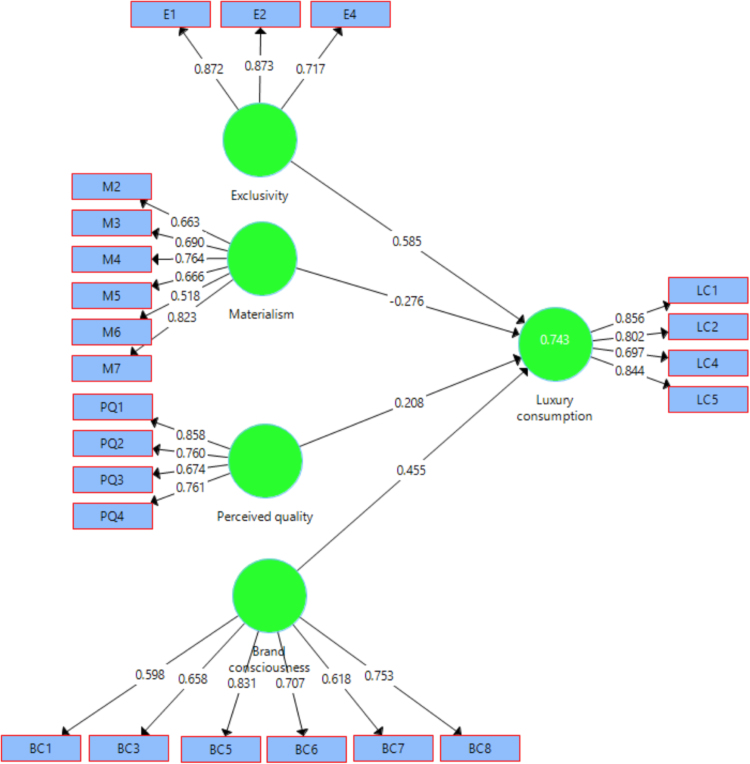
Measurement and structural model results.

Overall, *R*^2^ in Fig. 1 indicates that the research model explains 74.3% of the variance in luxury consumption. The following formula, provided by Tenenhaus et al. [5], the global goodness-of-fit (GoF) statistic for the research model was calculated using the equation:

$$\begin{array}{c} Goodness\;of\;fit=2\sqrt{average\;of\;all\;AVEs\;values*average\;of\;all{\;R}^{2}}\\ =2\sqrt{0.575*0.743}\\ =0.43 \end{array}$$

where AVE represents the average of all AVE values for the research variables while *R*^2^ represents the average of all *R*^2^ values in the full path model. The calculated global GoF is 0.43, which exceeds the suggested threshold of GoF > 0.36 suggested by Wetzels et al. [6]. Therefore, this data article concludes that the research model has a good overall fit.

### 2.2 Path model

The PLS estimation results for the structural model, path coefficients values as well as the item loadings for the research constructs are shown in Fig. 1 (Table 2).

**Table 2:** Outcomes of structural equation model analysis.

Path	Hypothesis	Path coefficients (β)	T- Statistics	Decision
Exclusivity -> Luxury consumption	H1(+)	0.585	7.456	Positive and significant
Materialism -> Luxury consumption	H2(+)	−0.276	1.946	Negative and insignificant
Perceived quality -> Luxury consumption	H3(+)	0.208	3.130	Positive and significant
Brand consciousness -> Luxury consumption	H5 (+)	0.455	3.360	Positive and significant

The primary source of data (questionnaire) was used for collecting data from a cross section of customers within the Cape Town metropolitan area. A Microsoft Excel spread sheet was used to enter all the data and to make inferences of the data obtained. The Statistical Packages for Social Sciences (SPSS) and the Smart PLS software for structural equation modelling (SEM) technique were used to code data and to run the statistical analysis [2]. Moreover, Smart PLS supports both exploratory and confirmatory research; it is robust to deviations for multivariate normal distributions and is good for a small sample size [2].

## 3 Ethical considerations

The researchers guaranteed that respondents were knowledgeable about the background and the aim of this research and they were kept well-informed with the participation process. Respondents were offered the chance to remain anonymous and their reactions were dealt with in confidence.

## 4 Academic, practical, and policy implications of this data article

The present data article offers implications for academicians. For instance, the data indicates that brand consciousness directly influences luxury consumption in a positive and significant way as indicated by a path coefficient of (β = 0.455). Therefore, for academicians in the field of marketing, this discovery enhances their understanding of the relationship between brand consciousness and luxury consumption as this is a useful contribution to the existing literature on these two variables. On the practitioners’ side, this data article submits that marketing managers can benefit from the implications of these discoveries. For example, given the robust relationship between exclusivity and luxury consumption (β = 0.585), marketing managers ought to pay attention or they should put more emphasis on product innovation as well as selling unique products since exclusivity acts as a facilitator of luxury consumption. Moreover, the present data article offers implications for policy makers who have been developing consumer business policies that enhance luxury consumption. Policies that exist in various retail institutions can be modified to incorporate luxury consumption. Thus, the discoveries obtained from this study׳s data set may be used to generate new policies and assist in the revision of existing policies.
