# Supplementary material for: Data to model the prognosticators of luxury consumption: A partial least squares-structural equation modelling approach (PLS-SEM)
Source: Data Brief. 2018 Oct 15;21:753–7. doi: 10.1016/j.dib.2018.10.032 (PMC6214865; doi:10.1016/j.dib.2018.10.032)
Supplement: Supplementary file 1 — Supplementary material [file mmc1.docx]

The authors hereby declare that there is no conflict of interest related to this data article
